# Prevalence and risk factors of significant persistent pain symptoms after critical care illness: a prospective multicentric study

**DOI:** 10.1186/s13054-023-04491-w

**Published:** 2023-05-25

**Authors:** Alexandre Bourdiol, Vincent Legros, Fanny Vardon-Bounes, Thomas Rimmele, Paul Abraham, Clément Hoffmann, Claire Dahyot-Fizelier, Maud Jonas, Pierre Bouju, Cédric Cirenei, Yoann Launey, Gregoire Le Gac, Samia Boubeche, Edouard Lamarche, Olivier Huet, Lucillia Bezu, Julie Darrieussecq, Magdalena Szczot, Agathe Delbove, Johan Schmitt, Sigismond Lasocki, Johann Auchabie, Ludivine Petit, Emmanuelle Kuhn-Bougouin, Karim Asehnoune, Hugo Ingles, Antoine Roquilly, Raphaël Cinotti, the ALGO-RÉA study group, the Atlanréa Group, Amélie Yavchitz, Stéphanie Sigault, Aurélien Mazereaud, Lucilia Bezu, Maxime Léger, Jean-Noël Evain, the Société Française d’Anesthésie-Réanimation–SFAR Research Network

**Affiliations:** 1 Pôle Anesthésie Réanimations, Service d’Anesthésie Réanimation chirurgicale, Hôtel Dieu, Nantes Université, CHU Nantes, 44093 Nantes, France; 2 Service d’Anesthésie–Réanimation, Hôpital Maison Blanche, CHU de Reims, 51100 Reims, France; 3 Service d’Anesthésie–Réanimation, Hôpital Rangueil, CHU de Toulouse, Toulouse, France; 4 Service d’Anesthésie-Réanimation, Hôpital Edouard Herriot, Hospices Civils de Lyon, Lyon, France; 5 EA7426 Pathophysiology of Injury-Induced Immunosuppression (Pi3), Hospices Civils de Lyon-Biomérieux-Université Claude Bernard Lyon 1, Lyon, France; 6 Service de médecine Intensive Adulte, Centre Hospitalier Universitaire Vaudois, Lausanne, Switzerland; 7 Burn Center, Percy Military Training Hospital, 101, Avenue Henri Barbusse - BP 406, 92141 Clamart, France; 8 Intensive Care and Anesthesia Department, University Hospital of Poitiers, University of Poitiers, Poitiers, France; 9 INSERM U1770, University of Poitiers, Poitiers, France; 10 Service de Réanimation, Hôpital de Saint-Nazaire, Saint-Nazaire, France; 11 Service de Réanimation Polyvalente, Centre Hospitalier de Bretagne Sud, Lorient, France; 12 Hôpital Claude Huriez, Pôle Anesthésie-Réanimation, médecine périopératoire et douleur, CHU Lille, 59000 Lille, France; 13 Department of Anaesthesia and Critical Care, Pontchaillou, University Hospital of Rennes, Rennes, France; 14 UMR_S 1242, Chemistry Oncogenesis Stress Signaling, University of Rennes, 35000 Rennes, France; 15 Service d’Anesthésie–Réanimation, CHU de Rouen, Rouen, France; 16 Department of Anaesthesia and Critical Care, University Hospital of Tours, 37000 Tours, France; 17 Department of Anaesthesia and Critical Care, University Hospital of Brest, 29000 Brest, France; 18 Service de Réanimation Polyvalente, Gustave Roussy, 94805 Villejuif, France; 19 Metabolomics and Cell Biology Platforms, Université Paris Saclay, Université de Paris, Sorbonne Université, Inserm UMR1138, Villejuif, France; 20 CH Aubagne, Pôle CARK, Service d’Anesthésie-Réanimation chirurgicale, Edmond Garcin, 179 Av. des soeurs Gastine, 13400 Aubagne, France; 21 Service d’Anesthésie-Réanimation, Hôpital Hautepierre, CHU Strasbourg, Strasbourg, France; 22 Service de Réanimation Polyvalente, CHBA Vannes, Vannes, France; 23 Hôpital d’Instruction des Armées Clermont Tonnerre, Rue Colonel Fonferrier, 29240 Brest, France; 24 Department of Anaesthesia and Critical Care, University Hospital of Tours, 49100 Angers, France; 25 Service de Réanimation, centre hospitalier de Cholet, Cholet, France; 26 CHU Saint-Etienne, Service d’Anesthésie-Réanimation, Saint-Étienne, France; 27 Centre d’Etude et de Traitement de la Douleur, Hôtel Dieu, Nantes Université, CHU Nantes, 44093 Nantes, France; 28 UMR 1064, Center for Research in Transplantation and Translational Immunology, INSERM, Nantes Université, 44000 Nantes, France; 29 MethodS in Patients-Centered Outcomes and HEalth Research, SPHERE, INSERM, Nantes Université, Univ Tours, CHU Nantes, CHU Tours, 44000 Nantes, France; 30 Department of Anesthesia and Critical Care, Hôtel-Dieu, University Hospital of Nantes, 1 place Alexis Ricordeau, 44093 Nantes, France

**Keywords:** Pain, Neuropathic pain, ID-pain, Critical care, Post-intensive care syndrome

## Abstract

**Background:**

Prevalence, risk factors and medical management of persistent pain symptoms after critical care illness have not been thoroughly investigated.

**Methods:**

We performed a prospective multicentric study in patients with an intensive care unit (ICU) length of stay ≥ 48 h. The primary outcome was the prevalence of significant persistent pain, defined as a numeric rating scale (NRS) ≥ 3, 3 months after admission. Secondary outcomes were the prevalence of symptoms compatible with neuropathic pain (ID-pain score > 3) and the risk factors of persistent pain.

**Results:**

Eight hundred fourteen patients were included over a 10-month period in 26 centers. Patients had a mean age of 57 (± 17) years with a SAPS 2 score of 32 (± 16) (mean ± SD). The median ICU length of stay was 6 [4–12] days (median [interquartile]). At 3 months, the median intensity of pain symptoms was 2 [1–5] in the entire population, and 388 (47.7%) patients had significant pain. In this group, 34 (8.7%) patients had symptoms compatible with neuropathic pain. Female (Odds Ratio 1.5 95% CI [1.1–2.1]), prior use of anti-depressive agents (OR 2.2 95% CI [1.3–4]), prone positioning (OR 3 95% CI [1.4–6.4]) and the presence of pain symptoms on ICU discharge (NRS ≥ 3) (OR 2.4 95% CI [1.7–3.4]) were risk factors of persistent pain. Compared with sepsis, patients admitted for trauma (non neuro) (OR 3.5 95% CI [2.1–6]) were particularly at risk of persistent pain. Only 35 (11.3%) patients had specialist pain management by 3 months.

**Conclusions:**

Persistent pain symptoms were frequent in critical illness survivors and specialized management remained infrequent. Innovative approaches must be developed in the ICU to minimize the consequences of pain.

*Trial registration.* NCT04817696. Registered March 26, 2021.

**Supplementary Information:**

The online version contains supplementary material available at 10.1186/s13054-023-04491-w.

## Background

Chronic pain is a major public health issue. On an individual level, chronic pain is associated with significant impairment of quality of life and major societal costs with loss of work productivity [1]. Several social, psychological, and genetic factors have been associated with chronic pain [2]. Beyond neuronal pain, the mechanisms involved in the development of chronic pain—immune mediators released by the central nervous system from astrocytes or infiltrating T-cells have been reported to modulate pain [3]. Moreover, neuropathic pain has been identified as a potential neuroimmune disorder [4]. However, the crosstalk between inflammatory pathways and the development of chronic pain is complex and acute inflammatory responses are not necessarily linked with chronic pain [5]. These phenomena underline the complexity of the pathophysiology behind the transition from acute to chronic pain. Critically ill patients undergoing numerous nociceptive interventions with significant procedural pain [6] are prescribed numerous analgesics (continuous morphine, remifentanil, ketamine) or immune-modulatory drugs (corticoids) and present major inflammatory disorders [7]. As stated by previous authors [8, 9], critically ill survivors are at risk of chronic pain but there is still a major gap of knowledge on this topic. We performed a prospective multicentric study to explore the epidemiology of persistent pain symptoms 3 months after ICU admission.

## Methods

The ALGO-REA study was a prospective multicentric longitudinal study (Additional file 1: STROBE statement) involving 26 Intensive Care Units (ICU) in university and other hospitals from April 2021 to January 2022 in France (NCT04817696). The study was approved by an Ethics Committee (Comité pour la Protection des Personnes Sud-Est III, N° 2021-019 B). Patients received oral and written information prior to enrolment and provided informed consent to participate.

### Inclusion criteria

All patients aged ≥ 18 years old, with an ICU length of stay ≥ 48 h and in whom pain symptoms were assessable on ICU discharge were eligible for this study. Pain assessment was left to the attending physician's discretion. Participating hospitals screened and included patients over a minimum period of 3 months and performed follow-up. We included in the final analysis patients with complete follow-up at 3 months.

### Exclusion criteria

Exclusion criteria were patients under 18 years old, lost to follow-up, pregnancy or breastfeeding, under guardianship or trusteeship, inability to evaluate pain on ICU discharge and refusal to participate.

### Exploration of pain symptoms and follow-up

On ICU discharge, investigators asked patients to quantify potential pain symptoms with a numerical rating scale (NRS) and completed the ID-Pain score along with anatomical location of symptoms in order to screen symptoms compatible with neuropathic pain [10]. Diagnosis of neuropathic pain requires a physical evaluation. Since follow-up was remotely performed, we used the ID-Pain score which can be validated in this context for screening [10]. Three-month follow-up was performed by each center via phone and/or snail mail so patients could complete a self-assessment questionnaire. The choice of follow-up method was at the discretion of each center based on local resources. Patients were asked to assess pain symptoms in the previous week before the evaluation. We asked patients to evaluate the intensity of their potential pain symptoms with an NRS. The symptoms compatible with neuropathic pain were evaluated with the ID-Pain score. We also explored whether patients were treated with analgesics (paracetamol, non-steroidal anti-inflammatory drugs, tramadol, morphine, anti-hyperalgesia drugs) and follow-up by a pain specialist (yes or no).

### Primary outcome

Primary outcome was the rate of persistent pain defined as a pain score with an NRS ≥ 3/10, 3 months after ICU admission. Patients with an NRS < 3 were classified as having no or mild pain.

### Secondary outcomes

Secondary outcomes were the intensity of pain symptoms on ICU discharge evaluated via NRS, the characteristics of symptoms compatible with neuropathic pain on discharge and at 3 months after admission assessed via ID-Pain score [10], evolution at 3 months of patients with or without significant pain symptoms on ICU discharge, anatomical localization of pain symptoms focusing on 6 regions (head, abdomen, thorax, back, limbs, joints) [10], ICU management of patients with and without chronic pain, and the nature of analgesics prescribed at 3 months. Finally, we explored the risk factors associated with persistent pain symptoms.

### Data collection

The following baseline characteristics were collected: gender, age, weight, height, comorbidities (stroke, ischemic cardiomyopathy, active smoking, hypertension, chronic obstructive pulmonary disease, diabetes, history of cancer, alcohol intake, dyslipidemia, history of depression) and chronic medication before admission [anti-depressive agents, type of analgesics, anti-hyperalgesia drugs (antidepressant tricyclic agent, pregabalin or gabapentin)]. We collected the simplified acute physiology score (SAPS II) and the reason for admission: traumatic brain injury, trauma without brain injury, burn, major cardio-thoracic surgery, sepsis/septic shock, COVID-19, acute respiratory failure not related to COVID-19, other major surgery and other causes of admission (miscellaneous). Regarding ICU management, we collected the following data: the type and total number of surgical interventions (neurosurgery, spine, orthopedics, abdominal, thoracic, insertion of thoracic drainage), use of continuous intravenous morphine, use of continuous intravenous ketamine, use of continuous intravenous remifentanil, use of antihyperalgesic drugs, use of continuous intravenous local anesthetic agents such as intravenous xylocaine and the use of loco-regional anesthesia techniques. We also recorded general complications such as the occurrence of acute respiratory distress syndrome, prone positioning, the use of neuromuscular blocking agents, the duration of invasive mechanical ventilation and length of ICU stay.

### General ICU care

All patients were treated according to international guidelines and local protocols. Patient analgesia and sedation management in the ICU, according to previously validated scales (Richmond assessment sedation scale, behavior pain scale, for example [11]) and guidelines [12, 13]. The use of analgesics on discharge (paracetamol, non-steroidal anti-inflammatory medication, oral morphine) was left to the attending physician’s discretion. Pain management on discharge (medication and follow-up) was performed according to local standards.

### Statistical analysis

Patients with persistent pain defined as NRS ≥ 3 at 3 months were compared with patients without persistent pain (NRS < 3). Numeric variables were expressed as mean (± SD) or median [1^st^–3^rd^ quartile] and discrete outcomes as absolute and relative (%) frequencies. We compared baseline demographic and follow-up data between groups with or without significant pain symptoms. Normality and heteroskedasticity of continuous data were assessed with Shapiro–Wilk and Levene’s tests, respectively. Continuous outcomes were compared with unpaired Student's *t*-test or Mann–Whitney U test according to data distribution. Discrete outcomes were compared with chi-squared or Fisher’s exact tests accordingly. The alpha risk was set at 5% and two-tailed tests were used. Based on our local experience of post-ICU follow-up during which we screened approximately 20% of the patients with pain at 6 months (unpublished data), we intended to include and perform follow-up in 700 patients. We therefore assumed that 140 patients would display persistent pain in order to perform a relevant multivariable logistic regression analysis and explore the risk factors of persistent pain 3 months after discharge. First, the groups with or without persistent pain were compared in univariate analysis. A specific model was elaborated according to the parsimonious rule. Variables associated with persistent pain in univariate analysis with a *p* value ≤ 0.15 were kept in the model. A backward selection process was then applied to elaborate the final model. Since we collected multiple causes of admissions, we pooled medical causes, surgical causes, and left trauma and burn patients alone. Sepsis was pooled in medical cause of admission and was set as the reference in the model owing to previously published data [14]. We selected a model with significant *p* values, not according to the overall model fit. Data were checked for multicollinearity with the Belsley-Kuh-Welsch technique. A *p* value < 0.05 was considered statistically significant. Patients with missing data were excluded from the analysis. Statistical analysis was performed with the online application EasyMedStat (version 3.19; www.easymedstat.com).

## Results

**Fig. 1:**
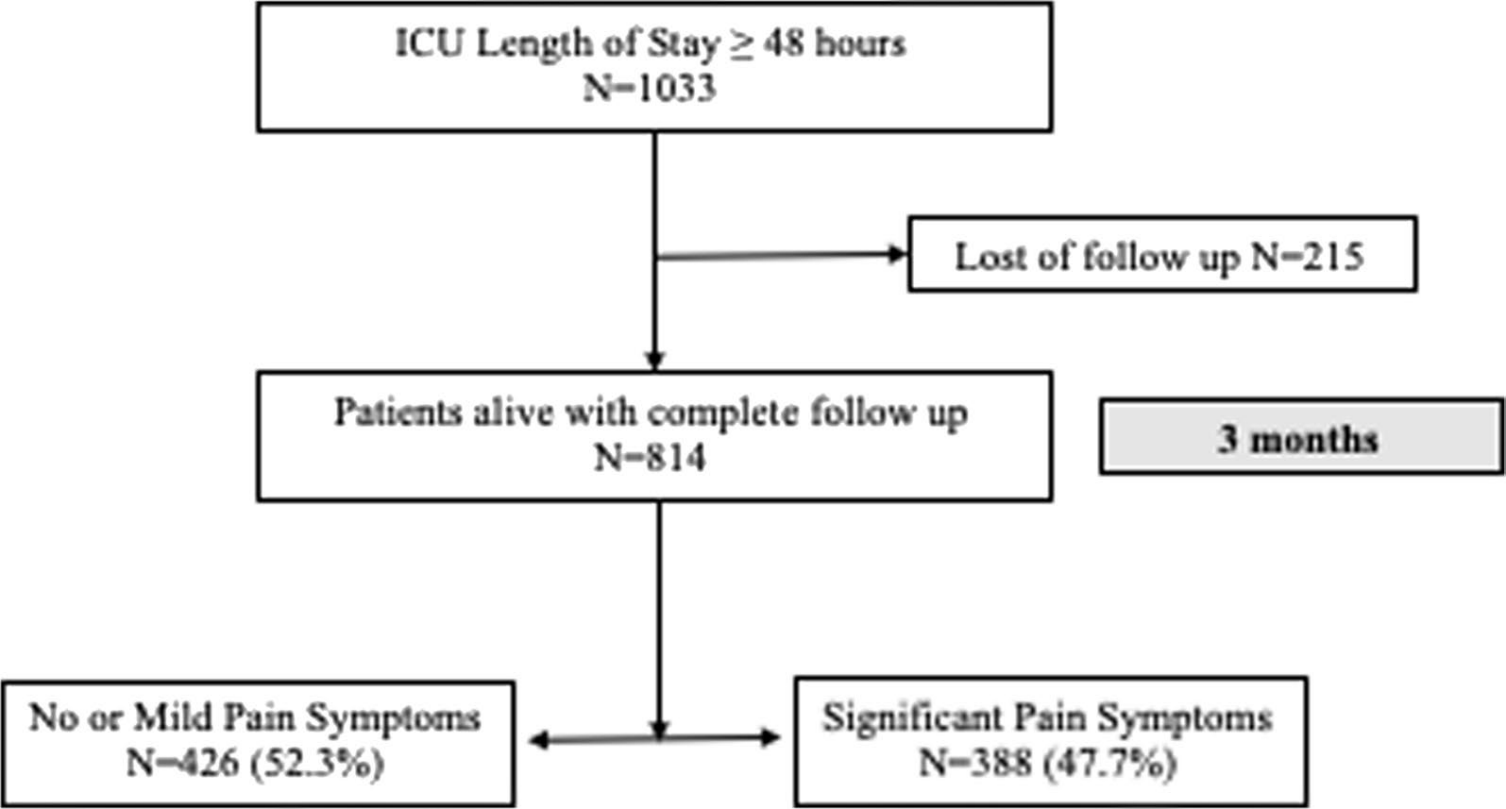
Flowchart of the study

The study was performed from April 2021 to January 2022 and 1,033 patients were included in 26 ICUs. After exclusion of 215 (20.8%) patients lost to follow-up at 3 months, 814 patients were included in the analysis. Mean age was 57 (± 17) years and 544 (66.5%) patients were male (Fig. 1). Patients were hospitalized for major non-thoracic surgery (207 (25.3%) patients), trauma (non neuro) (125/814, 15.3% patients), COVID-19 (74/814, 9.1% patients), sepsis (72/814, 8.8% patients), and major thoracic surgery (67/814, 8.2% patients). Before admission, 90 (11%) patients had chronic medication with paracetamol or non-steroidal anti-inflammatory drugs and 72 (9%) patients had antidepressant drugs. Patients had a median duration of invasive mechanical ventilation of 0 [0–2] days and a duration of ICU length of stay of 6 [4–12] days. Baseline characteristics are available in Table 1. Patients lost to follow-up were significantly older, had significantly more history of stroke and chronic obstructive pulmonary disease and had more chronic medication with paracetamol. There were no significant differences regarding ICU management, but patients lost to follow-up had a significantly shorter duration of ICU stay (5 [4–9] vs. 6 [4–12] days, *p* = 0.01) (Additional file 2: Table S1).

**Table 1:** Baseline characteristics of 814 patients with and without significant persistent pain at 3 months

	No or mild symptoms *N* = 426	Significant pain symptoms *N* = 388	*P *value
*Gender*			
Male	301 (70.8%)	240 (62%)	0.01
Female	124 (29.2%)	147 (38%)	
Age	58 (± 17)	56 (± 17)	0.2
Height (cm)	171 (± 13)	170 (± 10)	0.1
Weight (kg)	79 (± 19)	81 (± 21)	0.5
*Medical history*			
Hypertension	180 (42.3%)	152 (39.2%)	0.4
Chronic obstructive pulmonary disease	34 (8%)	34 (8.8%)	0.8
Ischemic cardiomyopathy	57 (13.4%)	34 (8.8%)	0.05
Diabetes mellitus	78 (18.3%)	58 (15%)	0.2
History of cancer	91 (21.4%)	74 (19.1%)	0.5
Stroke	12 (2.8%)	12 (3.1%)	0.9
Hypercholesterolemia	79 (18.5%)	61 (15.7%)	0.3
Anxiety and depression syndrome	28 (6.6%)	40 (10.3%)	0.1
Active smoking	109 (25.7%)	102 (26.5%)	0.9
Chronic alcohol intake	62 (14.7%)	66 (17.1%)	0.4
*Chronic medication*			
Paracetamol, NSAID	44 (10.4%)	46 (11.9%)	0.6
Tramadol	14 (3.3%)	19 (4.9%)	0.3
Morphine	15 (3.5%)	19 (4.9%)	0.4
Gabapentin, Pregabalin	19 (4.5%)	25 (6.5%)	0.3
Neuroleptics	22 (5.2%)	30 (7.8%)	0.2
Antidepressant agent	27 (6.4%)	45 (11.8%)	0.01
*Cause of admission*			< 0.001
Traumatic brain injury	7 (1.6%)	8 (2%)	
Stroke	7 (1.6%)	7 (1.8%)	
Trauma (non neuro)	37 (8.7%)	87 (22.4%)	
Major thoracic surgery	37 (8.7%)	30 (7.7%)	
Burn	42 (9.9%)	23 (5.9%)	
Sepsis	51 (12%)	21 (5.4%)	
COVID-19	36 (8.5%)	37 (9.5%)	
Acute respiratory failure	29 (6.8%)	28 (7.2%)	
Non-thoracic surgery	116 (27.2%)	89 (22.9%)	
Other	64 (15%)	58 (15%)	

Chronic pain symptoms were defined as a Numeric Rating Scale ≥ 3, at 3-months after admission. NSAID: Non-Steroidal Anti-Inflammatory Drug. Numeric variables are expressed as mean (± SD). Continuous outcomes were compared with Student's *t*-test. Discrete outcomes were compared with chi-squared or Fisher’s (£) exact test

### Primary outcome

At 3 months, the median intensity of pain symptoms was 2 [1–5] in the entire population. At 3 months, 388 (47.7%) patients presented persistent pain, defined as a NRS ≥ 3. There were 284 (34.9%) patients with NRS ≥ 4. In univariate analysis, patients with persistent pain were significantly more frequently female [147 (38%) vs. 124 (29.2%), *p* = 0.01], had less history of ischemic cardiomyopathy [34 (8.8%) vs. 57 (13.4%), *p* = 0.05], had more history of anxiety and depression symptoms [40 (10.3%) vs. 28 (6.6%), *p* = 0.07] and more chronic antidepressant agent medication [45 (11.8%) vs. 27 (6.4%), *p* = 0.01]. There were no significant differences in the use of analgesics before admission.

### Secondary outcomes

On ICU discharge, 492 (60.4%) patients presented pain (NRS ≥ 3) and 32 (3.9%) patients presented symptoms compatible with neuropathic pain. Among these 492 patients, 279 (57.1%) presented persistent pain at 3 months. In the group of 326 patients without significant pain on ICU discharge, 109 (33.6%) presented persistent pain at 3 months. Among the 388 patients with persistent pain, 279 (71.9%) presented NRS ≥ 3 on ICU discharge.

Regarding ICU characteristics, patients with persistent pain experienced significantly more trauma (non neuro) [87 (22.4%) vs. 37 (8.7%), *p* < 0.001], underwent more frequently orthopedic [55 (14.2%) vs. 23 (5.4%), *p* < 0.001] and spinal surgery [19 (4.9%) vs. 4 (0.9%), *p* = 0.001]. There were no other significant differences between groups regarding ICU management (Table 2).

**Table 2:** Management in the intensive care unit and characteristics on discharge

	No or mild symptoms at 3 months *N* = 426	Significant symptoms at 3 months *N* = 388	*P* value
SAPS 2	33 (± 16)	32 (± 16)	0.8
*Surgery during ICU stay*			
Intra-cranial	19 (4.5%)	17 (4.4%)	> 0.9
Spinal	4 (0.9%)	19 (4.9%)	0.001
Orthopaedics	23 (5.4%)	55 (14.2%)	< 0.001
Intra-abdominal	132 (31%)	107 (27.6%)	0.3
Thoracic	64 (15%)	50 (12.3%)	0.4
Pleural drainage	32 (7.5%)	34 (8.7%)	0.6
Number of interventions during ICU	1 [0–1]	1 [0–1]	0.6
*Pain management*			
Continuous Morphine	196 (46%)	170 (43.8%)	0.6
Remifentanil	41 (9.6%)	51 (13.1%)	0.1
Continuous Ketamine	49 (11.5%)	61 (15.8%)	0.1
Gabapentin, Pregabalin	42 (9.9%)	50 (12.9%)	0.2
Loco-regional anesthesia	84 (19.7%)	98 (25.3%)	0.1
*Pain symptoms on discharge*			
Numeric Rating Scale	2 [1–4]	4 [2 –6]	< 0.001
ID pain score	0 [0–1]	1 [0–2]	< 0.001
ID pain score > 3	10 (2.3%)	22 (5.6%)	0.02
*Pain location*			
Head	26 (6.1%)	33 (8.5%)	0.2
Abdominal	105 (24.7%)	101 (26%)	0.8
Limb	61 (14.3%)	86 (22.1%)	0.005
Joint	27 (6.3%)	29 (7.5%)	0.6
Thorax	59 (13.9%)	75 (19.3%)	0.04
Back	23 (5.4%)	60 (15.5%)	< 0.001
*ICU complications*			
Acute respiratory distress syndrome	45 (10.6%)	39 (10.1%)	0.9
Prone positioning	19 (4.5%)	29 (7.5%)	0.1
Neuro-muscular blocking agents	54 (12.7%)	48 (12.5%)	0.9
Invasive mechanical ventilation duration (days)	0 [0–2]	0 [0–2]	0.5
ICU length of stay (days)	6 [4–12]	6 [4–11]	0.9

Numeric variables are expressed as mean (± SD) or median [1^st^–3^rd^ quartile]. Numeric variables were expressed as mean (± SD). Continuous outcomes were compared with Student's *t*-test. Discrete outcomes were compared with chi-squared or Fisher’s (£) exact test

*SAPS* Simplified Acute Physiological Score, *ICU* Intensive Care Unit

In the group of patients with significant persistent pain, 34 (8.7%) patients had an ID-Pain score > 3, which stresses symptoms compatible with neuropathic pain, and specific pain specialist follow-up was performed in 35 (11.3%) patients. The characteristics of pain at 3 months are available in Table 3.

**Table 3:** Characteristics of significant persistent pain at 3-months after admission in the intensive care unit

	No or mild symptoms *N* = 426	Significant pain symptoms *N* = 388	*P* value
*Pain characteristics at 3 months*			
Numeric rating scale	1 [1–1]	5 [3–6]	–
ID pain score	0 [0–0]	1 [0–2]	< 0.001
ID pain score > 3	3 (0.7%)	34 (8.7%)	< 0.001
*Pain location*			
Head	3 (0.7%)	32 (8.3%)	< 0.001
Abdomen	15 (3.5%)	89 (22.9%)	< 0.001
Limb	22 (5.2%)	118 (30.4%)	< 0.001
Joint	9 (2.1%)	73 (18.8%)	< 0.001
Thorax	13 (3.1%)	73 (18.8%)	< 0.001
Back	7 (1.6%)	69 (17.8%)	< 0.001
*Pain killers at 3 months*			
Paracetamol, NSAID	57 (14.9%)	206 (66.5%)	< 0.001
Nefopam, Tramadol	9 (2.3%)	65 (21%)	< 0.001
Morphine	4 (1.1%)	37 (12%)	< 0.001
Gabapentin, Pregabalin	16 (4.5%)	46 (14.9%)	< 0.001
Neuroleptics	34 (9.5%)	42 (13.4%)	0.1
Specialist pain management	10 (2.61%)	35 (11.3%)	< 0.001

We did not detail the location of pain symptoms regarding upper limb versus lower limb. Numeric variables are expressed as median [1^st^–3^rd^ quartile]. Continuous outcomes were compared with Student's *t*-test. Discrete outcomes were compared with chi-squared or Fisher’s (£) exact test

*NSAID* Non-Steroidal Anti-Inflammatory Drug, *ICU* Intensive Care Unit

In the multivariable analysis, the risk factors significantly associated with persistent pain were female (Odds Ratio 1.5 95% Confidence Interval [1.1–2.1], *p* = 0.02), the use of anti-depressive agents before admission (OR 2.2 95% CI [1.3–4], *p* = 0.006), prone positioning (OR 3 95% CI [1.4–6.4], *p* = 0.003), the intensity of pain on ICU discharge (NRS ≥ 3) (OR 2.4 95% CI [1.7–3.4], *p* < 0.0001). Compared with medical causes of admission, patients admitted for trauma (non neuro) (OR 3.5 95% CI [2.1–6], *p* < 0.0001) were particularly at risk of persistent pain. Table 4 presents the risk factors of persistent pain by 3 months after ICU admission.

**Table 4:** Risk factors of significant persistent pain symptoms, 3 months after ICU admission

Risk factors	*N* =	Odds ratio	95% Confidence interval	*P *value
Female	270	1.5	[1.1–2.1]	0.02
Anti-depressive agents	72	2.2	[1.3–4]	0.006
Prone positioning in the ICU	47	3	[1.4–6.4]	0.003
NRS ≥ 3 on ICU discharge	483	2.4	[1.7–3.4]	< 0.0001
*Cause of admission*				
Medical cause	199	Ref		
Trauma (non neuro)	123	3.5	[2.1–6]	< 0.0001
Surgical cause	265	1.1	[0.8–1.7]	0.5
Burn	65	1.04	[0.5–1.9]	0.9

*ICU* Intensive Care Unit, *NRS* Numeric Rating Scale

## Discussion

In our prospective multicentric cohort, we found that almost half of the patients experienced significant pain 3 months after admission in an ICU. Among them, roughly 8% presented symptoms compatible with neuropathic pain and 11% had specialized follow-up.

Pain symptoms have been little described after critical care admission. In a review**,** Kemp et al. [8] noted that in the year following admission in an ICU, the prevalence of pain symptoms ranged from 14 to 77%. However, the data regarding pain symptoms were mostly extracted from randomized controlled trials not designed on the management of pain: these trials focused on enteral nutrition, mechanical ventilation, delirium [8]… Moreover, some studies used dedicated scales such as the Brief Pain Inventory but most studies used general scales not specifically targeting the analysis of pain symptoms (Short Form Health Survey, EuroQol-5D) [8]. In a retrospective monocentric cohort performed in 323 patients [14], Battle et al. evaluated the prevalence of chronic pain symptoms from 6 months to 1 year after discharge using the Brief Pain Inventory. The authors reported that 44% of patients presented chronic pain and identified age and sepsis as risk factors. In another monocentric cohort [15], the authors found that approximately 20% of patients presented chronic pain and that half of them had neuropathic pain. Finally, a monocentric study with a 1-year follow-up found a prevalence of pain symptoms of 49% in survivors at 3 months and pain symptoms compatible with chronic pain in 38% of survivors [16]. In an international cohort including more than 40,000 individuals across Europe [17], the authors evaluated that 19% suffered from pain symptoms. Our results and previously published studies suggest that survivors of critical illness present a greater prevalence of pain symptoms. Since all evaluations of pain symptoms were remotely performed, the exact link between critical illness and pain symptoms could be difficult to establish. Finally, the findings suggest that pain symptoms could be the consequence of critical illness, ICU management but also that survivors could be more sensitive to diverse miscellaneous pain stimuli and pain symptoms are extremely common [15, 16, 18].

We upheld the diagnosis of persistent pain with an NRS ≥ 3, but our definition is questionable. In the context of postoperative pain, a threshold ≥ 4 is commonly used [19], but some authors have used an NRS of 3 [20], or the occurrence of a new pain symptom whatever the intensity (NRS > 0, [21]). In the context of critical care illness, Battle et al. did not specify the intensity of pain symptoms [14]. Koster-Brouwer [15] and Langerud et al. [16] upheld the diagnosis classified patients with and without chronic pain, according to a yes/no question. Since there is no consensus about the adequate threshold that should be used to screen for patients with significant pain symptoms after critical care illness, we selected NRS ≥ 3 which appeared clinically relevant to potentially start specific treatment for pain. Moreover, we chose to screen pain symptoms after 3 months. This could appear somewhat fast in comparison with others, but there is currently no consensus on the time-points to perform screening of post-intensive care syndrome. Our idea was that a quick screening could allow early treatment and thus mitigate the risk of chronic pain. Overall, the exact rate of pain symptoms and their risk factors could differ across studies and a consensus on the definition and time-points of evaluation would provide consistency to further studies on this topic.

The risk factors of chronic pain after critical illness deserve cautious interpretation to this day. As stated before, the definitions and time-point of evaluation are not congruent across studies [14–16]. Moreover, some studies were retrospective [14, 16] and used cross-sectional designs [9]. Accounting for confounders could therefore be challenging. Nevertheless, pre-existing psychological issues such as anxiety and depression have already been identified as risk factors for post-intensive care syndrome [8] which is in line with our findings. Other authors found that sepsis was a risk factor for chronic pain [14, 15]. We also confirm that pain symptoms are common after sepsis but stress that trauma patients are even at higher risk. After traumatic brain injury, some authors have described that headache could be present in more than half of the patients, pain in other sites could be present in 40% of the patients and musculoskeletal pain could be present in more than 80% of the patients after 15 years [22]. In spite of this common issue after trauma, there is still much to do to fill the gap of knowledge and propose innovative approaches to improve outcomes.

Females presented significantly more symptoms than males. Koster-Brouwer et al. also found that females were at higher risk [15]. A possible explanation for this finding could be that females are administered higher doses of opioids whose exposure is known to induce chronic pain [8] compared with males in respect of their body weights. Finally, we identified prone positioning as a risk factor. We assume that this position would stretch joints such as shoulders which is a common site of pain symptoms in our study and others [14, 23].

The transition from acute to chronic pain is a complex phenomenon which involves an interaction between the host inflammatory response and the central nervous system [3]. There are several hypotheses behind the pathophysiology of the transition from acute to chronic pain, involving G protein-coupled receptor kinase with a desensitization of receptor secondary to inflammation, or the influence of the host immune response with transcriptomic or epigenetic modifications of the microglia [3]. Recently, Parisien et al. [5] demonstrated in a cohort of 98 patients with chronic low back pain, that patients in the group with resolved pain had thousands of transcriptomic changes, none of which were identified in the group of patients with chronic pain. They identified that an upregulation of the neutrophil-driven inflammatory response was protective against chronic pain. In an experimental mice model of pain [5], the authors demonstrated the causal implication of this neutrophil-driven inflammatory response on the development of chronic pain. Moreover, both in patients and the experimental study, the authors [5] showed that in spite of an early efficacy of steroids or non-steroidal anti-inflammatory drugs, these medications were associated with an increased risk of chronic pain. These findings illustrate the complexity of the transition from acute to chronic pain. Given the numerous nociceptive stimuli, the massive administration of medications with potential hyperalgesia effects such as opioids [3], and the complexity of the host immune response [7, 24], critically ill patients seem to be at high risk of developing post-ICU pain. Our results tend to support our primary hypothesis that all types of admissions were associated with the development of chronic pain. Moreover, the lack of relevant clinical risk factors identified in the present study and by previous authors [15, 18] underlines that the identification of high-risk patients could require in-depth biological analysis. Finally, these high-risk patients could benefit from a more proactive management of acute and procedural pain during ICU stay. Indeed, the large prevalence of patients displaying pain on discharge could emphasize shortcomings in our care for acute pain during the hospitalization.

## Limitations

We did not use the Brief Pain Inventory in our study. However, owing to the lack of a gold standard, we used an NRS in this evaluation which has been largely used in the surgical context. Also, we did not perform face-to-face interviews to assess pain symptoms for logistic reasons. A face-to-face versus a remote evaluation could influence the evaluation of pain symptoms [25]. Given the paucity of data in the literature on this topic, we chose to perform remote evaluations in order to facilitate the recruitment and evaluation of patients. Further cohorts with face-to-face interviews could be interesting after critical care illness, but such a design would be highly challenging in terms of feasibility. Furthermore, we did not standardize the time of the day when pain symptoms were assessed. Again, given the paucity of data on this topic, we chose to simplify the screening and monitoring of patients during follow-up. More stringent evaluation could clearly alter our findings but again a more demanding follow-up would enhance pain evaluation but could also increase the number of patients lost to follow-up which was already high in our cohort (20%) in spite of easy and achievable post-ICU screening. Some items such as the socio-cultural backgrounds of patients which are known to influence pain symptoms, the time-frame of administration of medications of interest (anti-depressive agents, pain killers), or the presence of myositis ossificans during follow-up were not collected in our study. Since the evaluation was remotely performed, we could not properly evaluate that pain symptoms were post-ICU persistent or specific to other pain stimuli that occurred during the evaluation. However, these characteristics could be challenging even face-to-face. For instance, joint pain could be directly related to mispositioning in the ICU but could appear not to be linked with the reason for admission (intra-abdominal surgery, for instance). Since we included patients who could assess their pain symptoms, we induced a selection bias by not considering patients with neurological issues (delirium, brain injury). The underlying idea was to see whether pain symptoms on discharge could be an early marker of persistent symptoms but patients with impaired consciousness could also be at risk after discharge. We did not perform missing data imputation, since the primary aim of the study was to explore the prevalence of pain symptoms. Finally, we chose the ID-Pain score to screen for symptoms compatible with neuropathic pain. Although it is less specific than the DN-4 score, it is sensible to screen symptoms compatible with neuropathic pain since it provides anatomical localization and is meant for remote evaluation [10].

## Conclusions

Persistent pain could be a common health problem after critical illness care. Our data also suggest that there is a lack of specific follow-up after ICU discharge. Given the major consequences on patient quality of life, the social implications and health-care costs, it appears to be important to design innovative interventions to minimize the consequences of pain after critical care illness.

## Supplementary Information

**Additional file 1:** STROBE checklist.

**Additional file 2: Table S1.** Univariate analysis of patients with and without lost to follow-up.

**Additional file 3:** The ALGO-RÉA study group.

## Data Availability

All data generated or analyzed during this study are included in this published article as Additional files.

## Abbreviations

ICU: Intensive Care Unit
SAPS: Simplified acute physiological score
NSAID: Non-steroidal anti-inflammatory drug
