# Supplementary material for: Prevalence and risk factors of significant persistent pain symptoms after critical care illness: a prospective multicentric study
Source: Crit Care. 2023 May 25;27:199. doi: 10.1186/s13054-023-04491-w (PMC10208914; doi:10.1186/s13054-023-04491-w)
Supplement: Supplementary file 1 — Additional file 1: STROBE checklist. [file 13054_2023_4491_MOESM1_ESM.docx]

STROBE Statement—checklist of items that should be included in reports of observational studies

|  | Item No | Recommendation |
| --- | --- | --- |
| **Title and abstract** | 1 | (*a*) Indicate the study’s design with a commonly used term in the title or the abstract. P1 |
|  |  | (*b*) Provide in the abstract an informative and balanced summary of what was done and what was found. P3 |
| Introduction | | |
| Background/rationale | 2 | Explain the scientific background and rationale for the investigation being reported. P4 |
| Objectives | 3 | State specific objectives, including any prespecified hypotheses. P4 |
| Methods | | |
| Study design | 4 | Present key elements of study design early in the paper. P5-6 |
| Setting | 5 | Describe the setting, locations, and relevant dates, including periods of recruitment, exposure, follow-up, and data collection. P5 |
| Participants | 6 | (*a*) *Cohort study*—Give the eligibility criteria, and the sources and methods of selection of participants. Describe methods of follow-up. P5-6 |
|  |  | (*b*) *Cohort study*—For matched studies, give matching criteria and number of exposed and unexposed. P7-8 |
| Variables | 7 | Clearly define all outcomes, exposures, predictors, potential confounders, and effect modifiers. Give diagnostic criteria, if applicable. P7-8 |
| Data sources/ measurement | 8* | For each variable of interest, give sources of data and details of methods of assessment (measurement). Describe comparability of assessment methods if there is more than one group. P 6-7 |
| Bias | 9 | Describe any efforts to address potential sources of bias. NA |
| Study size | 10 | Explain how the study size was arrived at. P8-9 |
| Quantitative variables | 11 | Explain how quantitative variables were handled in the analyses. If applicable, describe which groupings were chosen and why. P8-9 |
| Statistical methods | 12 | (*a*) Describe all statistical methods, including those used to control for confounding  P8-9 |
|  |  | (*b*) Describe any methods used to examine subgroups and interactions P8-9 |
|  |  | (*c*) Explain how missing data were addressed NA |
|  |  | (*d*) *Cohort study*—If applicable, explain how loss to follow-up was addressed  P 5 |
|  |  | (*e*) Describe any sensitivity analyses NA |

Continued on next page

| Results | | |
| --- | --- | --- |
| Participants | 13* | (a) Report numbers of individuals at each stage of study—eg numbers potentially eligible, examined for eligibility, confirmed eligible, included in the study, completing follow-up, and analysed. P9 |
|  |  | (b) Give reasons for non-participation at each stage NA |
|  |  | (c) Consider use of a flow diagram. NA |
| Descriptive data | 14* | (a) Give characteristics of study participants (eg demographic, clinical, social) and information on exposures and potential confounders. P9, Table 1 |
|  |  | (b) Indicate number of participants with missing data for each variable of interest. P9, Supplemental Table 1 |
|  |  | (c) *Cohort study*—Summarise follow-up time (eg, average and total amount) NA |
| Outcome data | 15* | *Cohort study*—Report numbers of outcome events or summary measures over time. P9-10, Table 1 and 2 |
| Main results | 16 | (*a*) Give unadjusted estimates and, if applicable, confounder-adjusted estimates and their precision (eg, 95% confidence interval). Make clear which confounders were adjusted for and why they were included. P10, Table 4 |
|  |  | (*b*) Report category boundaries when continuous variables were categorized NA |
|  |  | (*c*) If relevant, consider translating estimates of relative risk into absolute risk for a meaningful time period NA |
| Other analyses | 17 | Report other analyses done—eg analyses of subgroups and interactions, and sensitivity analyses. Supplementary Information |
| Discussion | | |
| Key results | 18 | Summarise key results with reference to study objectives. P11 |
| Limitations | 19 | Discuss limitations of the study, taking into account sources of potential bias or imprecision. Discuss both direction and magnitude of any potential bias. P12 |
| Interpretation | 20 | Give a cautious overall interpretation of results considering objectives, limitations, multiplicity of analyses, results from similar studies, and other relevant evidence P13 |
| Generalisability | 21 | Discuss the generalisability (external validity) of the study results. P13 |
| Other information | | |
| Funding | 22 | Give the source of funding and the role of the funders for the present study and, if applicable, for the original study on which the present article is based. P2 |

*Give information separately for cases and controls in case-control studies and, if applicable, for exposed and unexposed groups in cohort and cross-sectional studies.
