# Supplementary material for: Prevalence and risk factors of significant persistent pain symptoms after critical care illness: a prospective multicentric study
Source: Crit Care. 2023 May 25;27:199. doi: 10.1186/s13054-023-04491-w (PMC10208914; doi:10.1186/s13054-023-04491-w)
Supplement: Supplementary file 2 — Additional file 2: Table S1. Univariate analysis of patients with and without lost to follow-up. [file 13054_2023_4491_MOESM2_ESM.docx]

**Supplementary Table 1.** Univariate analysis of patients with and without lost to follow-up.

|  | Patients with follow-up  N = 823 | Lost to follow-up  N = 255 | *P value* |
| --- | --- | --- | --- |
| **Gender** Male Female | 549 (66.9%) 272 (33.1%) | 167 (67.3%) 81 (32.6%) | 0.9 |
| Age | 57 (± 17) | 60 (± 17) | 0.03 |
| Height (cm)  Weight (kg)  **Medical history** Hypertension | 171 (±11) 79 (±20)  337 (41%) | 171 (±10) 76 (±18)  117 (45.9%) | 0.9 0.004  0.2 |
| Chronic Obstructive Pulmonary Disease | 69 (8.4%) | 38 (14.9%) | 0.003 |
| Ischemic cardiomyopathy | 93 (11.3%) | 31 (12.2%) | 0.8 |
| Diabetes mellitus | 139 (16.9%) | 45 (17.7%) | 0.9 |
| History of cancer | 169 (20.5%) | 66 (25.9%) | 0.08 |
| Stroke | 25 (3%) | 18 (7.1%) | 0.007 |
| Hypercholesterolemia | 145 (17.6%) | 36 (14.1%) | 0.2 |
| Anxiety and depression syndrome | 68 (8.3%) | 34 (13.3%) | 0.02 |
| Active smoking | 212 (26%) | 67 (26.9%) | 0.8 |
| Chronic alcohol intake | 130 (15.9%) | 49 (19.8%) | 0.2 |
| **Chronic medication** Paracetamol, NSAID | 91 (11.1%) | 54 (22%) | <0.01 |
| Tramadol | 33 (4%) | 19 (7.7%) | 0.03 |
| Morphine | 34 (4.2%) | 18 (7.3%) | 0.07 |
| Anti-Hyperalgesia drug | 44 (5.4%) | 17 (6.9%) | 0.4 |
| Neuroleptics | 52 (6.3%) | 14 (5.7%) | 0.8 |
| Antidepressant agent | 73 (9%) | 27 (11%) | 0.4 |
| **Cause of Admission** Traumatic Brain Injury Stroke Trauma (non neuro) Major thoracic surgery  Burn  Sepsis  COVID-19  Acute Respiratory Failure  Other surgery  Other | 15 (1.8%)  14 (1.7%)  125 (15.2%)  67 (8.1%)  65 (7.9%)  74 (9%)  74 (9%)  57 (6.9%)  209 (25.4%)  123 (15%) | 8 (3.2%)  1 (0.4%)  36 (14.5%)  12 (4.8%)  3 (1.2%)  25 (10%)  18 (7.2%)  34 (13.7%)  51 (20.5%)  61 (24.5%) | <0.001 |
| SAPS 2 | 32 (± 16) | 35 (± 17) | 0.07 |
| **Pain management** Continuous Morphine | 368 (44.7%) | 98 (39.5%) | 0.2 |
| Remifentanil | 92 (11.2%) | 29 (11.7%) | 0.9 |
| Continuous Ketamine | 110 (13.4%) | 22 (8.9%) | 0.08 |
| Anti-Hyperalgesia drugs | 92 (11.2%) | 35 (14.1%) | 0.3 |
| Loco-regional anaesthesia | 183 (22.2%) | 44 (17.7%) | 0.2 |
| **ICU complications** Acute Respiratory Distress Syndrome | 85 (10.3%) | 20 (8.1%) | 0.4 |
| Prone positioning | 49 (6%) | 13 (5.3%) | 0.8 |
| Continuous neuro-muscular blocking agents | 103 (12.6%) | 22 (8.9%) | 0.1 |
| Invasive mechanical ventilation duration (days) | 0 [0–2] | 0 [0–1] | 0.08 |
| ICU length of stay (days) | 6 [4–12] | 5 [4–9] | 0.01 |

**Legend.** NSAID: Non-Steroidal Anti-Inflammatory Drug. SAPS: Simplified Acute Physiological Score. ICU: Intensive Care Unit
